# Glucocorticoids Alter CRTC-CREB Signaling in Muscle Cells: Impact on PGC-1α Expression and Atrophy Markers

**DOI:** 10.1371/journal.pone.0159181

**Published:** 2016-07-12

**Authors:** Jill A. Rahnert, Bin Zheng, Matthew B. Hudson, Myra E. Woodworth-Hobbs, S. Russ Price

**Affiliations:** 1 Renal Division, Department of Medicine, Emory University, Atlanta, Georgia, United States of America; 2 Atlanta Veterans Affairs Medical Center, Decatur, Georgia, United States of America; University of Louisville School of Medicine, UNITED STATES

## Abstract

Muscle wasting associated with chronic diseases has been linked to decreased expression of PGC-1α and overexpression of PGC-1α counters muscle loss. CREB, in conjunction with the CREB-regulated transcription coactivator (CRTC2), is a positive modulator of PGC-1α transcription. We previously reported that PGC-1α expression is decreased in skeletal muscle of diabetic rats despite a high level of CREB phosphorylation (i.e., activation), suggesting that CRTC2-CREB signaling may be dysregulated. In this study, the relationship between CREB/CRTC signaling and PGC-1α expression was examined in L6 myotubes treated with dexamethasone (Dex, 48h) to induce atrophy. Dex decreased PGC-1α mRNA and protein as well as the levels of CRTC1 and CRTC2 in the nucleus. Dex also altered the nuclear levels of two known regulators of CRTC2 localization; the amount of calcinuerin catalytic A subunit (CnA) was decreased whereas SIK was increased. To assess PGC-1α transcription, muscle cells were transfected with a PGC-1α luciferase reporter plasmid (PGC-1α-Luc). Dex suppressed PGC-1α luciferase activity while both isobutylmethylxanthine (IBMX) and over-expression of CRTC1 or CRTC2 increased PGC-1α-Luc activity. Mutation of the CRE binding site from PGC-1α-Luc reporter attenuated the responses to both IBMX and the CRTC proteins. Consistent with the reporter gene results, overexpression of CRTC2 produced an increase in CRTC2 in the nucleus and in PGC-1α mRNA and PGC-1α protein. Overexpression of CRTC2 was not sufficient to prevent the decrease in PGC-1α mRNA or protein by Dex. In summary, these data suggest that attenuated CREB/CRTC signaling contributes to the decrease in PGC-1α expression during atrophy.

## Introduction

Accelerated protein degradation contributes to the loss of skeletal muscle mass in a variety of catabolic conditions including sepsis, cancer cachexia, kidney disease and diabetes. Chronic systemic diseases (e.g., chronic kidney disease and diabetes) are often associated with prolonged increases in circulating glucocorticoids that contribute to protein degradation as adrenalectomy or treatment with glucocorticoid receptor antagonist attenuates muscle loss in these conditions [1–4]. These reports underscore the permissive role that glucocorticoids play in the activation of various proteolytic systems (e.g., ubiquitin-proteasome, autophagy) [5–9].

Muscle atrophy during chronic diseases has been linked to a decrease in the level of peroxisome proliferator-activated receptor gamma coactivator 1-alpha (PGC-1α) [10–14]. In skeletal muscle, PGC-1α is a transcriptional coactivator that regulates energy and other aspects of metabolism, in part, by facilitating a genetic program that drives the oxidative fiber phenotype, mitochondrial biogenesis, and fatty acid oxidation. In recent studies, overexpression of PGC-1α was reported to maintain muscle mass in several models of atrophy by a proposed mechanism that involves inhibition of the FoxO transcription factors. FoxO1 and FoxO3 are key regulators of a number of atrophy-related genes (i.e., atrogenes) [10,14–17]. Sandri et al [14] reported that overexpression of PGC-1α attenuated FoxO3a activity, thereby providing a protective effect against atrophy. In other studies, overexpression of PGC-1α prevented the induction of FoxO-mediated atrogenes, MuRF-1 and Atrogin-1/MAFbx, and the reduction in fiber size during atrophy [10,15]. Thus, maintenance of the level of PGC-1α in muscle appears to be important for sustaining muscle health and function.

The level of PGC-1α protein in cells is regulated by both transcriptional and post-transcriptional mechanisms. Consistent with its role as a “master” regulator, the PGC-1α promoter region has binding sites for a variety of transcription factors, thus providing inputs for various signaling pathways. Many of the contraction-induced metabolic adaptations are a result of increased PGC-1α expression that is mediated by MEF2 and CREB acting through their respective binding sites in the PGC-1α promoter. The CREB-regulated transcription coactivators (CRTCs) are a family of proteins that interact with phosphorylated CREB and enhance PGC-1α expression [18,19]. Among the 3 CRTC family members, CRTC1 mRNA is highly abundant in brain with low expression in other tissues while CRTC2 and CRTC3 mRNAs are ubiquitously expressed in most tissue including skeletal muscle [19]. In the nucleus, individual CRTCs form a complex with CREB and other proteins (e.g., p300) that interacts with cyclic AMP (cAMP) response elements [20,21]. Nuclear localization of the CRTCs is regulated by phosphorylation/dephosphorylation via the cAMP and calcium signaling pathways [22–24]. The phosphorylation of CRTCs by salt-inducible kinase (SIK) family members promotes their interactions with 14-3-3 proteins and sequestration in the cytosol. Nuclear localization of CRTCs is facilitated by cAMP-PKA mediated phosphorylation (inhibition) of SIK, or by CRTC dephosphorylation via calcineurin (Cn), a calcium-activated phosphatase.

We previously reported that a decrease in PGC-1α expression in muscle undergoing diabetes-induced atrophy was linked to attenuation of Cn activity [25]. This response occurred despite a high level of CREB phosphorylation (i.e. activation) [25]. These findings led us to posit that dysregulation of CRTC signaling during muscle atrophy could contribute to the reduced expression of PGC-1α during muscle wasting. This hypothesis was tested using cultured rat L6 myotubes treated with dexamethasone, a synthetic glucocorticoid, as a model of muscle atrophy. Responses to glucocorticoids in this model have been shown by our lab and others to recapitulate many features of the atrophy phenotype seen in rodents [2,16,26].

## Materials and Methods

### Cell Culture

Rat L6 myoblasts (American Type Culture Collection, Manassas, VA) were maintained in Dulbecco’s modified Eagles’s medium (DMEM, 25mM HEPES and 1.0 g/L glucose; Lonza, Walkersville, MD) supplemented with 10% fetal bovine serum (Atlanta Biologicals, Lawrencville, GA), 2% glutamine and 1% penicillin and streptomycin (pen-strep, Invitrogen, Carlsbad, CA). Differentiation into myotubes was induced by switching cells to DMEM supplemented with 2% horse serum, 2% glutamine and 1% pen-strep (differentiation media) for 3 days [27]. Following differentiation, myotubes were treated with dexamethasone (Dex, 100nM) for 48hrs with repeated treatment after 24hrs [26].

### Adenoviruses

In some experiments, L6 myotubes were infected at a moi of 25–50 with adenoviruses encoding GFP (gift from Dr. Franch, Emory University), human CRTC1 (Welgen, Worcester, MA) or human CRTC2 (Vector Biolabs, Philadelphia, PA) on day one or two of differentiation.

### Luciferase Activity

L6 myoblasts were plated in a 12-well plate and co-transfected with 30 ng of pRL-TS *Renilla* luciferase plasmid (to control transfection efficiency [28] and 300 ng of a firefly luciferase plasmid encoding the full 2Kb promoter region of PGC-1α (pPGC-1α-Luc, Addgene, Cambridge, MA) using FuGENE 6 (Roche Diagnostics, Indianapolis, IN) according to the manufacturer's instructions. To determine whether the cyclic AMP response element (CRE) site between -146 and -129 in the promoter region is important for DEX and other responses, some cells were transfected with a PGC-1α-luciferase plasmid similar to pPGC-1α-Luc except that the CRE site is mutated (pPGC-1α-ΔCRE-Luc, Addgene, Cambridge, MA). One day (24 h) after transfection, cells were infected with an adenovirus encoding GFP, CRTC1 or CRTC2 (25-50moi) with or without Dex (100nM) in differentiation media. After 48 h, firefly and *Renilla* luciferase activities were measured using the Dual Luciferase Reporter Assay System (Promega, Fitchburg, WI). In some experiments (as noted in Results), isobutylmethylxanthine (IBMX, 250μM) was added to the myotubes for 6 h prior to harvest and determination of luciferase activities. Normalized firefly luciferase activity (i.e. firefly:*Renilla*) was reported from 3–6 experiments consisting of 3–6 wells per treatment group/experiment. For statistical analysis, the mean treatment group values in each set of experiments were used and n = the number of times the experiment was repeated. In two cases with IBMX, a set of two experiments were performed to confirm the work of others. In these instances, there were 3 wells/treatment/experiment. Statistical analysis was performed on these experiments using individual wells and n = 6, the number of wells per treatment group.

### Cytosolic and Nuclear Fractionation

Cells were rinsed two times and scraped from culture dishes in ice-cold phosphate-buffered saline, followed by centrifugation at 1500×*g* for 5 min at 4°C. The pellet was resuspended in a solution containing 0.01 mol/L HEPES (pH 7.6–7.8), 1.5 mmol/L MgCl_2_, 2 mmol/L KCl, 0.5 mmol/L DTT, 0.5 mmol/L PMSF, 1% protease inhibitor cocktail (MiniComplete, Roche, Indianapolis, IN) and 1 mmol/L sodium orthovanadate and incubated on ice for 15 min. After the addition of Nonidet P-40 (final concentration 0.5%), samples were mixed vigorously for 10 s and centrifuged at 1500×*g* for 30–60 s at 4°C. The supernatant (cytosolic fraction) was transferred to a new tube and stored at −80°C until further analysis. The pellet was resuspended in a solution containing 0.02 mmol/L HEPES (pH 7.6–7.8), 1.5 mmol/L MgCl_2_, 2 mmol/L KCl, 0.4 mol/L NaCl, 0.2 mmol/L EDTA, 0.5 mmol/L DTT, 0.5 mmol/L PMSF, 1% protease inhibitor cocktail, 1 mmol/L sodium orthovanadate and 25% glycerol, and samples were incubated on a shaking platform for 15–60 min at 4°C. Samples were centrifuged at 21,000×*g* for 20 min at 4°C, and the supernatant (nuclear fraction) was transferred to a new tube and stored at −80°C until further analysis.

### Immunoblot Analysis

Cells were lysed in a buffer consisting of 50 mM HEPES (pH 7.5), 137 mM NaCl, 1 mM MgCl_2_, 1 mM CaCl_2_, 1 mM Na_3_VO_4_, 10 mM sodium pyrophosphate, 10 mM sodium fluoride, 2 mM EDTA, 1% Nonidet P-40, 10% glycerol supplemented with 1% protease inhibitor cocktail. Protein concentrations in cleared lysates were measured using a DC protein assay kit (BioRad Laboratories, Hercules, CA). Proteins were separated by SDS-PAGE, transferred to nitrocellulose membranes and detected by immunoblotting methods. Primary antibody dilutions were: PGC-1α 1:1000, pan Cn A catalytic subunit (CnA) 1:1000 (Millipore, San Diego, CA), CRTC1 1:1500, CRTC2 1:1500 and SIK1 1:1000 (Santa Cruz Biotechnology, Santa Cruz, CA). Antibodies to glyceraldehyde-3-phosphate dehydrogenase (GAPDH) and Histone H1 (Santa Cruz Biotechnology, Santa Cruz, CA, USA) were used to assess the purity of the nuclear and cytosolic samples. Unless otherwise stated in the figure legend, protein band intensities are quantified as the absolute band values and expressed as a percentage of the mean absolute control values for each experiment. Graphs depict the mean of all data. Equal loading and electroblot transfer of protein was verified by Ponceau S Red staining and imaging [26].

### RNA Isolation and Real-Time PCR

RNA was isolated using TRIzol (Invitrogen, Carlsbad, CA) and reverse transcribed using the Superscript III First-Strand Synthesis kit (Invitrogen, Carlsbad, CA) according to the manufacturer's instructions. mRNA was measured using quantitative real-time polymerase chain reaction (qPCR) with the BioRad iCycler and the iQ SYBR Green reagent (BioRad Laboratories, Hercules, CA, USA). Previously published primer set for PGC-1α [25] was used to perform PCR reactions. Sequences of primers were as follows for CRTC2 (forward) 5’-CTCTGCCCAATGTTAACCAGAT-3’, (reverse) 5’-GAGTGC TCCGAGATGAATCC-3’; for NR4A3 (forward) 5’-TCAGCCTTTTTGGAGCTGTT-3’, (reverse) 5’-GTCGAGCCA CTCCCCAAAT-3’; for TFam (forward) 5’-AAA TTG CAG CCA TGT GGA GG-3’, (reverse) 5’-CTC AGC TTT AAA ATC CGC TTC A-3’; for CytC (forward) 5’-TCA CCT GGG GAG AGG ATA CC-3’, (reverse) 5’-GGT CTG CCC TTT CTC CCT TC-3’; for Atrogin-1/MAFbx (forward) 5’-CAG AGC TGG GTG AAG ACG G-3’, (reverse) 5’-TAA CTG CTG AGG TCG CTC AC-3’; and for MuRF-1 (forward) 5’-GGA CGG AAA TGC TAT GGA GA-3’, (reverse) 5’-AAC GAC CTC CAG ACA TGG AC-3’. 18S was used as the normalization control. The data were analyzed for fold change (ΔΔCt) using the iCycler software, as previously described [26]. Melting curve analyses were performed to verify the specificity of the reaction.

### Statistical Analysis

All data are expressed as mean values ± standard deviations (s.d.) and statistical analyses were performed using Prism (GraphPad, La Jolla, CA). For one treatment, differences between groups were compared by two-tailed Student's *t* test. Differences were considered significant when p<0.05. When there were two treatments, differences among groups were compared by two-way ANOVA with a significance threshold of *p*<0.05 followed by *post hoc t*-tests using the Bonferroni-Dunn correction for multiple comparisons.

## Results

### Dexamethasone decreases PGC-1α gene and protein expression

**Fig 1:**
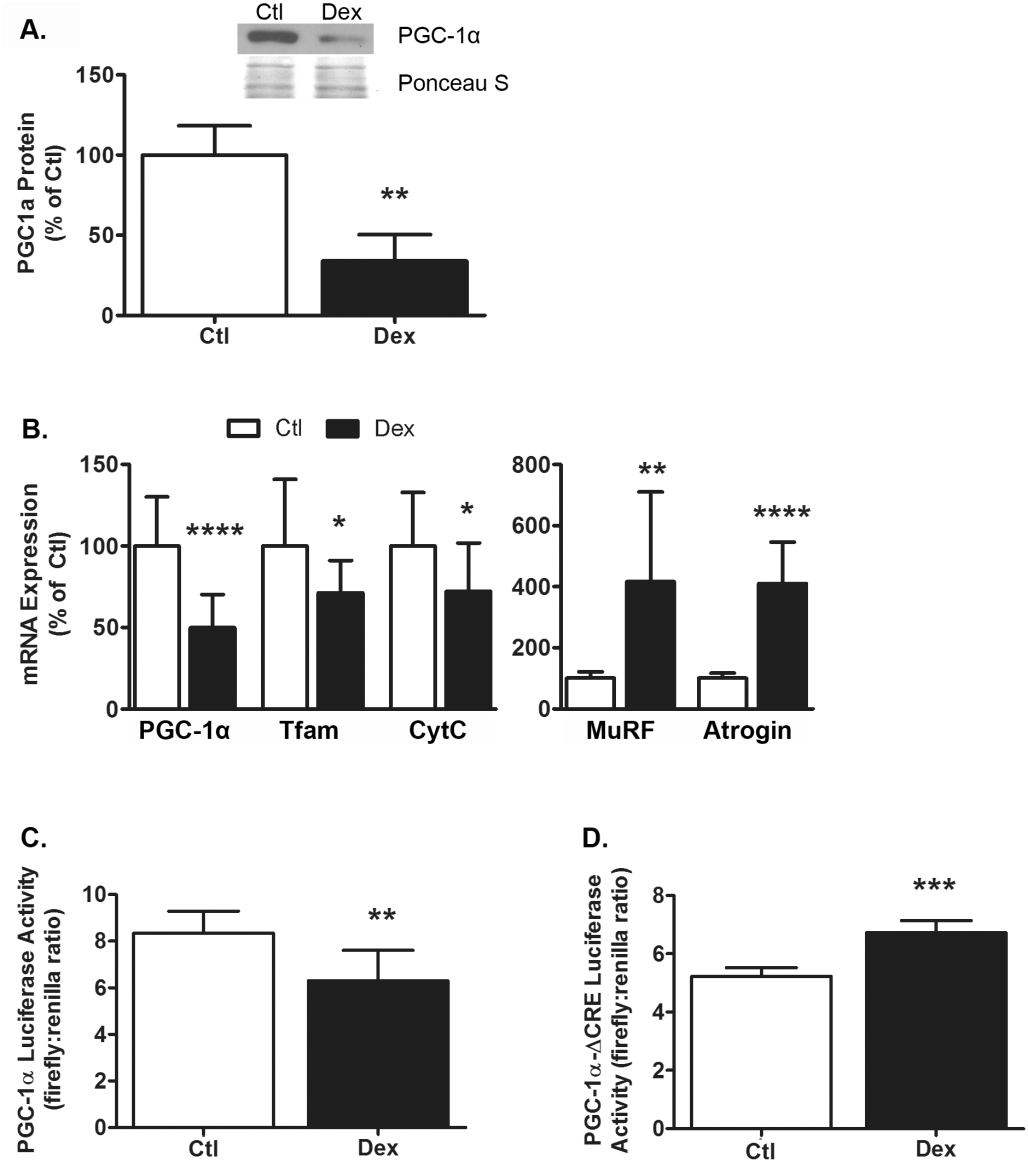
Dexamethasone decreases PGC-1α protein expression and transcription. (A) Treatment of L6 myotubes with Dex (100nM, 48hrs) decreased PGC-1α protein expression (p = 0.01). n = 3/treatment from 3 experiments. A representative western blot image and corresponding section of the Ponceau S—stained membrane are shown. (B) Dex decreased the mRNAs for PGC-1α (p<0.0001) and its target genes Tfam (p = 0.03) and CytC (p = 0.03); Dex also increased MuRF-1 (p = 0.003) and Atrogin-1/MAFbx mRNAs (p<0.0001). n = 10-14/treatment from 4–5 experiments. (C) In L6 cells transfected with a pPGC-1α-Luc, Dex decreased luciferase activity (p = 0.001). n = 6/treatment from 6 experiments. (D) In separate experiments with cells transfected with a pPGC-1α-ΔCRE-Luc, Dex increased luciferase activity (p = 0.0005). n = 3/treatment from 3 experiments. Data are expressed as mean values ± s.d. and were analyzed by Student’s *t*-test. Asterisks indicate values that are significantly different from control: p<0.05 = *, p<0.01 = **, p<0.001 = *** and p<0.0001 = ****.

Previously we reported PGC-1α mRNA and protein expression decreased in muscle during streptozotocin-induced diabetes [25]. To determine if glucocorticoids act as a signal that down-regulates PGC-1α, we tested whether the synthetic glucocorticoid dexamethasone (Dex) affects PGC-1α expression in L6 myotubes. Similar to diabetes, incubating myotubes with Dex for 48 h decreased PGC-1α protein by 66 ± 16% (p = 0.01, Fig 1A) and PGC-1α mRNA by 50 ± 20% (p = 0.0001, Fig 1B). Treatment with Dex also decreased the mRNAs for two PGC-1α-responsive genes; mitochondrial transcription factor A (Tfam) mRNA was reduced by 29 ± 20% (p = 0.03) and Cytochrome C (CytC) mRNA was lower by 28 ± 30% (p = 0.03, Fig 1B). Dex also increased the mRNAs for MuRF-1 and Atrogin-1/MAFbx, two well established atrogenes, by 416 ± 294% (p = 0.003) and 410 ± 135% (p<0.0001) respectively (Fig 1B). Next, we tested whether a decrease in PGC-1α transcription might contribute to the reduction in PGC-1α mRNA/protein and whether the response involves CREB. Myoblasts were transfected with one of two PGC-1α luciferase reporter plasmids: pPGC-1α-Luc contains the proximal ~2kb of the mouse PGC-1α promoter whereas pPGC-1α-Luc-ΔCRE has a mutation in the cAMP response element (CRE) site located between positions -146 and -129. In cells transfected with pPGC-1α-Luc, Dex decreased luciferase activity by 25 ± 13% (p = 0.0011, Fig 1C). In separate experiments, Dex did not decrease luciferase activity when cells were transfected with pPGC-1α-Luc-ΔCRE; instead, for reasons that are unclear, Dex increased luciferase activity by 28 ± 5% (p = 0.0005, Fig 1D). These data suggest that the reduction in PGC-1α expression occurs via a multi-faceted mechanism that includes inhibition of PGC-1α gene expression via the promoter CRE site as well as possibly other post-transcriptional responses.

### CREB activation and PGC-1α promoter activity

**Fig 2:**
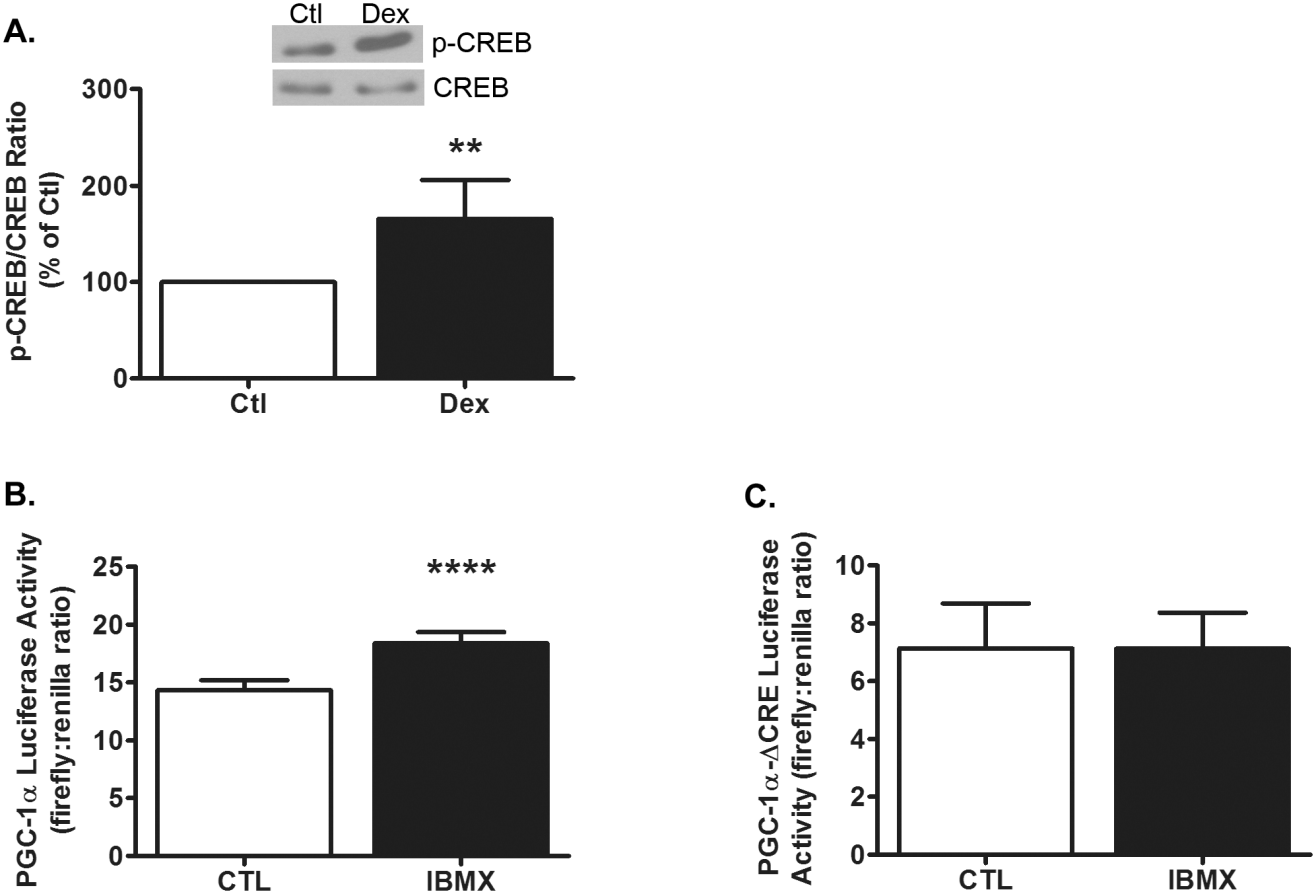
CREB activation and PGC-1α promoter activity. (A) Treatment of L6 myotubes with Dex (100nM, 48hrs) increased CREB phosphorylation relative to total CREB (p = 0.007). n = 5/treatment from 5 experiments. Representative western blot images of phospho-CREB and total CREB from a single experiment are shown above. (B) In L6 cells transfected with the pPGC-1α luciferase reporter plasmid, IBMX (250μM, 6 h) increased normalized luciferase activity (p<0.0001). n = 6/treatment from 2 experiments. (C) In separate experiments using cells transfected with a pPGC-1α-ΔCRE luciferase reporter plasmid, IBMX (250μM, 6 h) did not affect luciferase activity; n = 6/treatment from 2 experiments. Data are expressed as mean values ± s.d. and analyzed by Student’s *t*-test. Asterisks indicate a value that is significantly different from control: p<0.01 = ** and p<0.0001 = ****.

In normal cells, activation of CREB by raising cAMP is sufficient to increase PGC-1α expression [29]; however, in diabetic muscles undergoing atrophy, PGC-1α mRNA was decreased despite an increase in CREB phosphorylation [25]. Given these findings, we tested whether Dex induces CREB phosphorylation and found a 65 ± 40% increase in CREB phosphorylation in treated cells (p = 0.007, Fig 2A). Thus, the paradoxical increase in CREB phosphorylation and concomitant decrease in PGC-1α expression occur in both our myotube model and skeletal muscle in vivo. We next confirmed that activating CREB is sufficient to increase PGC-1α transcription in L6 myotubes under normal conditions that do not induce atrophy. Cells were first transfected with pPGC-1α-Luc or pPGC-1α-Luc-ΔCRE, followed by incubation with isobutylmethylxanthine (IBMX) for 6 h. IBMX is a phosphodiesterase inhibitor that raises intracellular cAMP, leading to the phosphorylation and activation of CREB. IBMX increased luciferase activity 28 ± 7% in cells transfected with pPGC-1α-Luc (p<0.0001, Fig 2B); in separate experiments using cells transfected with pPGC-1α-ΔCRE-Luc, the response did not occur (Fig 2C). These results suggest that under non-atrophy conditions, CREB regulation of PGC-1α via the CRE site is intact.

### Dexamethasone decreases CRTC1/CRTC2 nuclear localization

**Fig 3:**
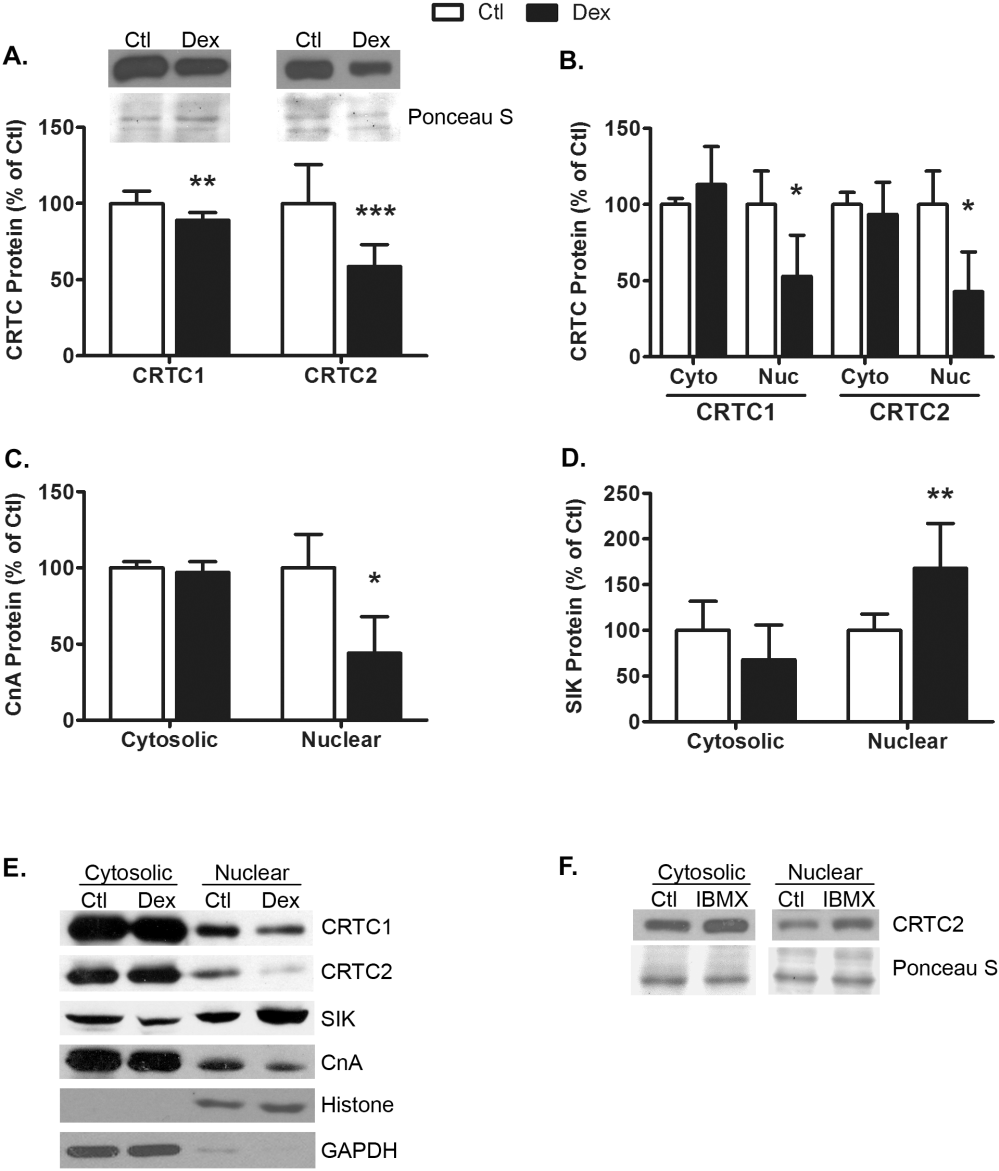
Dexamethasone decreases CRTC1 and CRTC2 nuclear localization. Treatment of L6 myotubes with Dex (100nM, 48hrs) decreased (A) total CRTC1 (p = 0.004 and CRTC2 protein (p = 0.0007). n = 9/treatment from 3 experiments. Representative western blots and corresponding sections of the Ponceau S-stained membranes are shown. Dex decreased (B) nuclear CRTC1 (p = 0.04) and nuclear CRTC2 (p = 0.03) protein and (C) nuclear CnA protein (p = 0.04); no differences in the cytosolic levels of these proteins were detected. Dex increased (D) nuclear SIK1 protein (p = 0.005) with no detectable difference in cytosolic protein. n = 3-7/treatment from 3–5 experiments. (E) Representative western blots for cytosolic and nuclear CRTC1, CRTC2, SIK, and CnA proteins are shown; controls for the nuclear (Histone) and cytosolic (GAPDH) fractions are also shown. (F) IBMX (250uM, 15 min) increased nuclear CRTC2 protein. A representative western blot image and corresponding Ponceau S-stained membrane from a single experiment run on the same gel are shown; the experiment was repeated one other time with similar results. In panels A-D, data are expressed as mean percent of control ± s.d and were analyzed by Student’s *t*-test. Asterisks indicate a value that is significantly different from control: p<0.05 = *, p<0.01 = ** and p<0.001 = ***.

In addition to phosphorylating CREB, PKA inactivates members of the SIK family of kinases that phosphorylate and inactivate CRTC1 and CRTC2 [23,24]. On the other hand, Cn, a calcium-activated phosphatase, supports the nuclear localization of CRTC proteins by dephosphorylating them. Therefore, we next examined whether Dex affects the localization of the CRTC proteins and their modulator proteins. The glucocorticoid decreased the total cellular content of both CRTC1 (11 ± 5%; p = 0.004) and CRTC2 (41 ± 14%; p = 0.0007) (Fig 3A). Similarly, the levels of CRTC1 and CRTC2 in the nuclear fraction were reduced 47 ± 27% (p = 0.04) and 57 ± 26% (p = 0.03), respectively, by Dex (Fig 3B and 3E). No significant changes in the cytosolic fractions of these proteins were detected. Consistent with the changes in CRTC1 and CRTC2 localization, Dex decreased the amount of nuclear Cn catalytic A subunit (CnA) by 57 ± 24% (p = 0.04) with no change in cytosolic CnA (Fig 3C and 3E). Conversely, Dex increased the amount of SIK1 by 68±49% (p = 0.005) with a trend for a reduction in cytosolic SIK1 (32 ± 38%, p = 0.1, Fig 3D and 3E). Lastly, treating control myotubes with IBMX increased nuclear CRTC2 by 37 ± 4% with no apparent change in cytosolic CRTC2 (Fig 3F), confirming that the CRTC translocation process is intact in untreated myotubes [23]. Altogether, these data support our hypothesis that Dex reduces the amount of CRTC1/CRTC2 in the nucleus by altering the levels of their modulators, SIK and CnA.

### Overexpression of CRTC2, Dex, and PGC-1α expression

**Fig 4:**
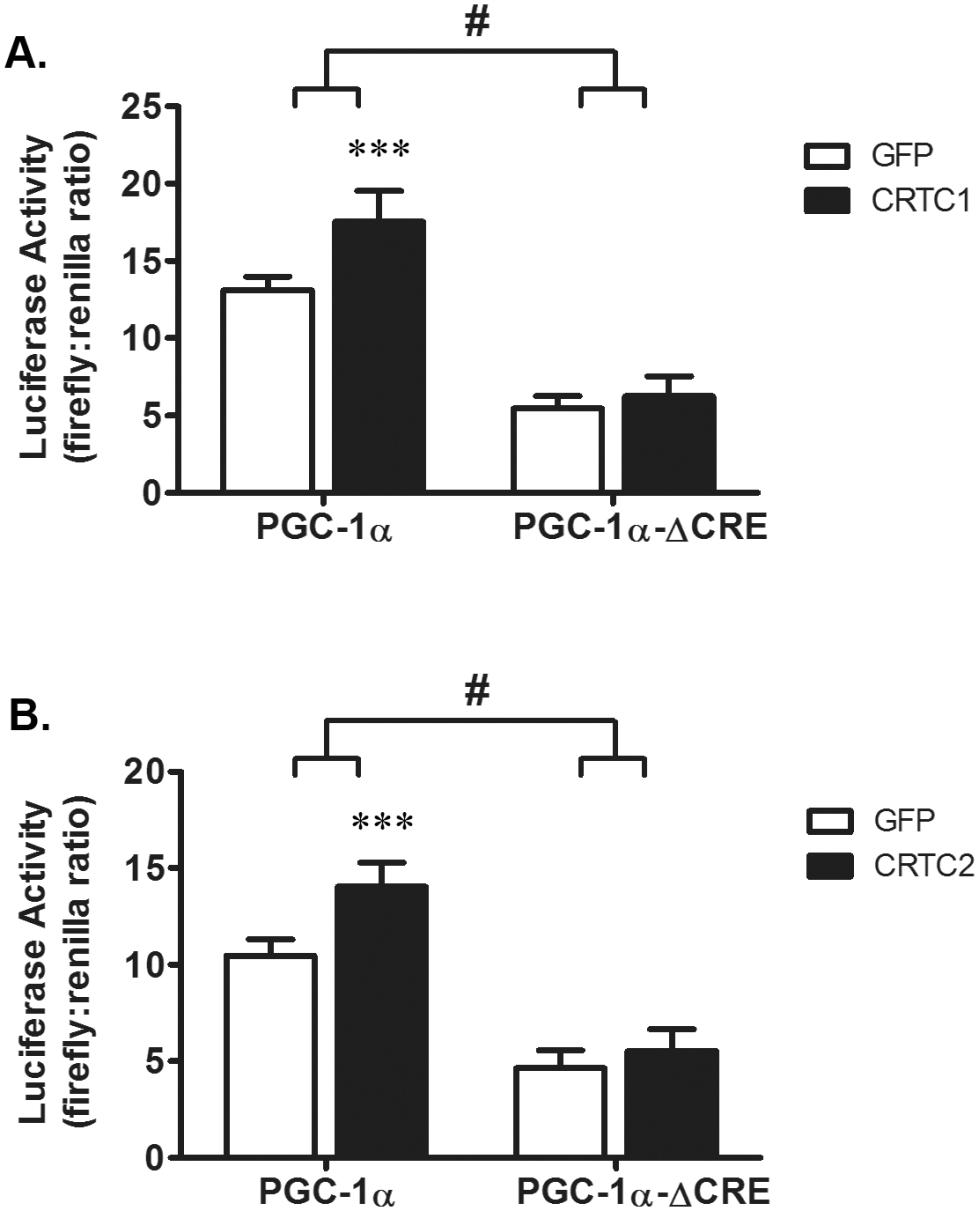
Overexpression of CRTC proteins increase PGC-1α transcription. L6 myoblasts were transfected with either pPGC-1α-Luc or pPGC-1α-ΔCRE-Luc. After 24 h, cells were infected with adenoviruses to express GFP, human CRTC1 or human CRTC2 and harvested 48 h later to measure luciferase activity. A two-way ANOVA was used to evaluate the significance of CRTC protein overexpression and the mutation of the CRE site on pPGC-1α-Luc activity. Overexpressing either CRTC1 (A) or CRTC2 (B) increased luciferase activity (CRTC1, p = 0.0001, CRTC2, p = 0.0002). In both sets of experiments, luciferase activity is reduced in cells transfected with pPGC-1α-ΔCRE-Luc (p<0.0001 in each panel). The CRTC1 or CRTC2-induced increase in luciferase activity was prevented by mutation of the CRE site in pPGC-1α-Luc (CRTC1, p = 0.003; CRTC2, p = 0.01). n = 5-6/treatment from 5–6 experiments. Data are expressed as the mean value ± s.d of normalized (i.e., firefly:renilla ratio) luciferase activity. The # indicates that the effects of CRTC1 or CRTC2 on luciferase activity are different at p<0.01 when CRE site is mutated in the PGC-1α promoter. Asterisks denote significant effects of CRTC1 or CRTC2 compared to GFP controls as indicated by *post hoc* analysis: p<0.001 = ***.

In previous reports, overexpression of CRTCs increased PGC-1α mRNA via a mechanism involving CREB [18,19]. To determine whether the CRTC-induced increase in PGC-1α promoter activity in L6 myotubes depends on a functional CRE site, we overexpressed human CRTC1 or CRTC2 in cells transfected with either the pPGC-1α-Luc or pPGC-1α-Luc-ΔCRE reporter plasmid and evaluated our results using a two-way ANOVA. Consistent with the previous reports, we found that PGC-1α luciferase activity increased in response to overexpression of either CRTC1 (34 ± 15%, Fig 4A) or CRTC2 (34 ± 12%, Fig 4B). When the CRE site was mutated, neither CRTC1 nor CRTC2 produced an increase in luciferase activity (Fig 4A and 4B). Moreover, the CRE site appears to be important for basal PGC-1α luciferase activity since luciferase activity was reduced 56–58%, depending on which CRTC protein was expressed, when the CRE site was mutated compared to luciferase activity when the CRE site is intact (p<0.0001). These data suggest that functional CRTC-CREB signaling, acting through the CRE site, is sufficient to induce PGC-1α promoter activity whereas dysfunctional CRTC-CREB signaling (e.g., no CRE) results in no induction.

**Fig 5:**
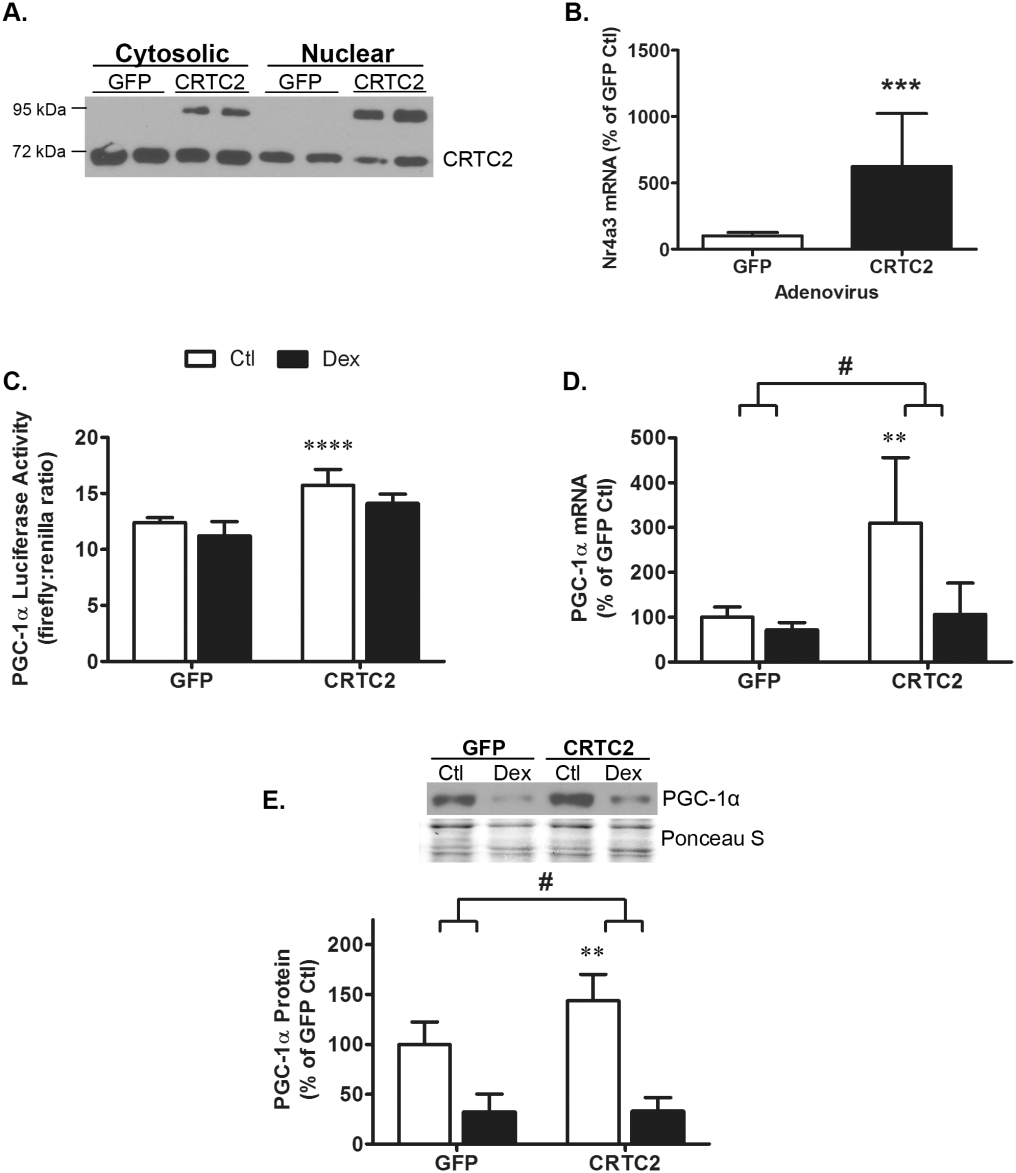
Dexamethasone, CRTC2 and PGC-1α expression. (A) A representative western blot confirms infection of L6 myotubes with virus encoding CRTC2 increases nuclear CRTC2 protein. Ectopic human CRTC2 protein runs as a ~90kDa protein while the endogenous protein is 67kDa. (B) Overexpression of human CRTC2 increased Nr4a3 mRNA expression as analyzed by Student’s *t*-test (p<0.001 = ***). n = 9/treatment from 3 experiments. (C, D, E) L6 myotubes were infected with GFP- or CRTC2-expressing adenovirus for 24 h then treated with or without 100nM Dex for 48 h. Two-way ANOVA was used to identify effects of CRTC2 overexpression and Dex treatment on PGC-1α luciferase activity, PGC-1α mRNA and PGC-1α protein. (C) PGC-1α luciferase activity increased with CRTC2 overexpression (p<0.0001) and decreased with Dex treatment (p = 0.006); ectopic CRTC2 did not prevent the reduction in luciferase activity with Dex (p = 0.6). n = 4/treatment from 4 experiments. (D) PGC-1α mRNA expression increased with CRTC2 overexpression (p = 0.0002) and decreased with Dex treatment (p = 0.0003). n = 8-9/treatment from 3 experiments. (E) PGC-1α protein increased with CRTC2 overexpression (p = 0.004) and decreased with Dex treatment (p<0.0001) n = 8-9/treatment from 3 experiments. A representative western blot image and corresponding Ponceau S-stained membrane is shown. Data are expressed as mean values ± s.d. The # indicates the effect of Dex on the measured outcome is different at p<0.01 when CRTC2 is overexpressed. Asterisks denote significant effects of CRTC2 compared to GFP controls as indicated by *post hoc* analysis: p<0.01 = ** and p<0.0001 = ****.

Having established that CRTC2 increases PGC-1α promoter activity, we further evaluated the functionality of the ectopic human CRTC2 protein. The virally-encoded protein (~90 kDa) is present in both the cytosol and nucleus (Fig 5A). Ectopic CRTC2 also increased the mRNA of NR4A3, a canonical gene target of CRTC/CREB signaling, by 623 ± 367% vs GFP-control cells (p = 0.0006, Fig 5B) [20,24]. We then evaluated whether overexpression of CRTC2 is sufficient to overcome the attenuating effects of Dex on PGC-1α expression. Consistent with the previous experiments (Fig 4A and 4B), CRTC2 overexpression induced pPGC-1α-Luc reporter activity by 27 ± 11% (Fig 5C); CRTC2 also increased PGC-1α mRNA by 310 ± 146% (Fig 5D) and increased PGC-1α protein by 44 ± 26% (Fig 5E). In contrast, overexpressing CRTC2 did not prevent the inhibitory effect of Dex on PGC-1α expression. Dex suppressed luciferase activity equally in cells infected with either the GFP or CRTC2 adenovirus (p = 0.6, Fig 5C). For unknown reasons, the Dex-induced decrease in luciferase activity is less pronounced in adenovirus-infected cells than in cells not infected with an adenovirus (i.e. Fig 1). Similarly, Dex reduced PGC-1α mRNA (p = 0.0003, Fig 5D) and PGC-1α protein (p = 0.0001, Fig 5E) in cells overexpressing either GFP or CRTC2. These data indicate that under normal, non-atrophic conditions, CRTCs contribute to the regulation of PGC-1α protein expression; however, increasing the level of CRTC2 in the nucleus is not sufficient to prevent the decline in PGC-1α expression under atrophic conditions.

### Overexpression of CRTC2, Dex, and atrogene expression

**Fig 6:**
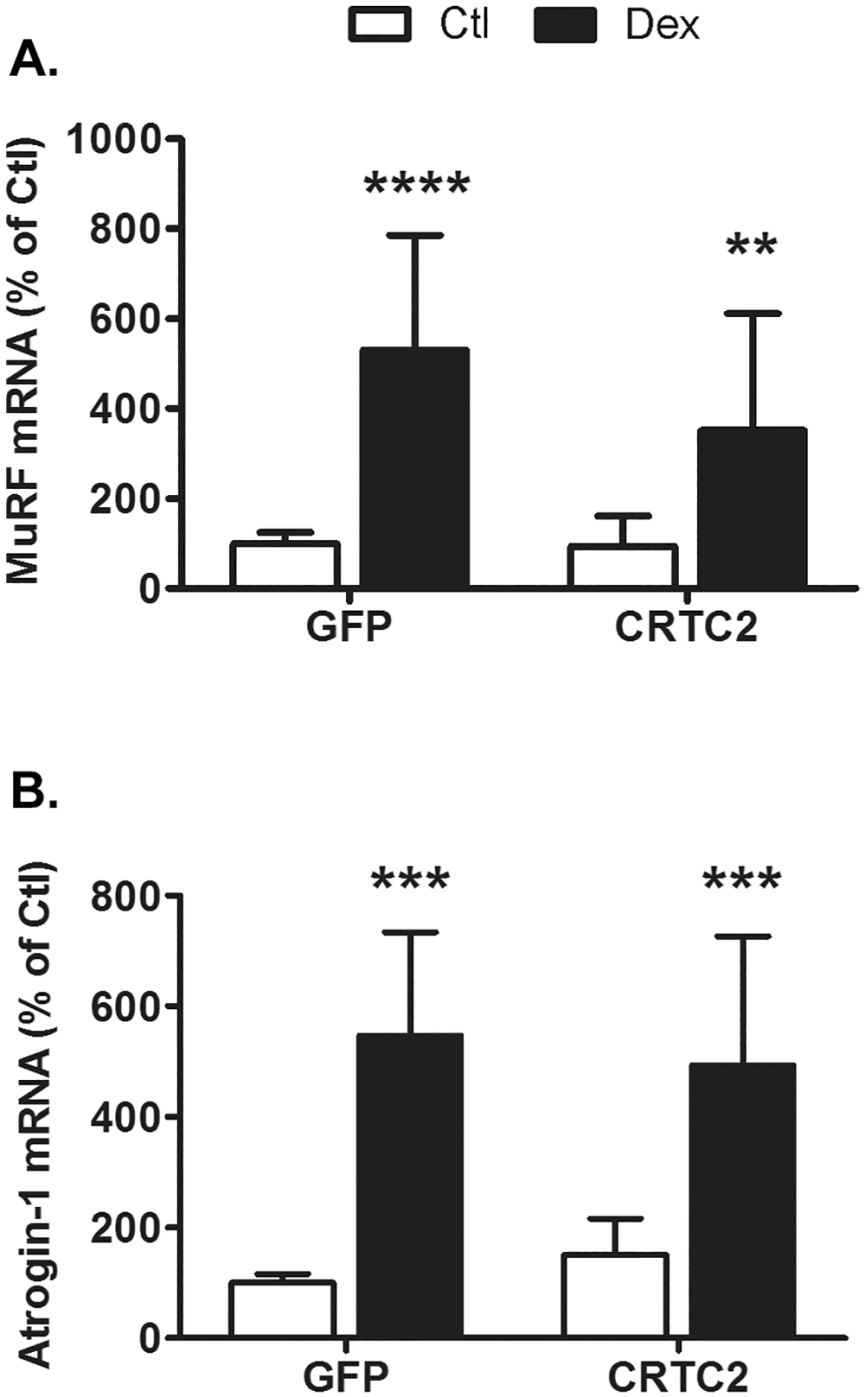
Dexamethasone, CRTC2, and atrogene expression. Two-way ANOVA was used to evaluate whether CRTC2 alters atrogene mRNA responses to Dex. Treatment with Dex increased (A) MuRF-1 mRNA (p<0.0001) and (B) Atrogin-1/MAFbx mRNA (p<0.0001). Overexpression of CRTC2 did not affect MuRF-1 or Atrogin/MAFbx mRNA expression (p≥0.1) or effect the response to Dex treatment (p≥0.1). n = 11-12/treatment from 4 experiments. Data are expressed as mean percent of control ± s.d. Asterisks denote significant effects of Dex compared to respective controls as indicated by *post hoc* analysis: p<0.01 = **, p<0.001 = *** and p<0.0001 = ****.

We and others have shown that glucocorticoids induce the expression of atrophy-related genes (e.g., Atrogin-1/MAFbx) via activation of the FoxO transcription factors [8,26]. Recently, PGC-1α was proposed to attenuate muscle protein loss by inhibiting FoxO-mediated atrophy signaling. [10,14,15]. Given this relationship, we next tested whether ectopic CRTC2 influences the induction of Atrogin-1/MAFbx and MuRF1 mRNAs by Dex. The results were evaluated by a two-way ANOVA. Dex increased the mRNA of MuRF-1 by greater than 352% (p<0.0001, Fig 6A) and Atrogin-1/MAFbx by greater than 492% (p<0.0001, Fig 6B) in cells expressing either CRTC2 or GFP; however, the magnitude of the induction by Dex was not different between cell groups (p≥0.12). These data indicate that increasing CRTC2 is not sufficient to prevent the Dex-induced reduction in PGC-1α or increase in the E3 ligase atrogenes.

## Discussion

PGC-1α is indicated to play an important role in the regulation of the FoxO-mediated transcription program that causes muscle atrophy. Expression of the coactivator is down-regulated in multiple models of atrophy and overexpression of PGC-1α can attenuate atrophy [10,14,15]. In our earlier studies with diabetic rats, PGC-1α in skeletal muscle was decreased despite a paradoxical increase in the phosphorylation of CREB, a known positive regulator of PGC-1α [25]. Given that optimal induction of PGC-1α transcription by CREB requires CRTC, we hypothesized that CRTC-CREB signaling might be dysregulated during glucocorticoid-induced atrophy and that these changes contribute to the reduced expression of PGC-1α. The primary findings related to this hypothesis are: 1) the CRE-site in the PGC-1α promoter is important for both basal PGC-1α promoter activity and the Dex-induced reduction in promoter activity; 2) Dex decreases nuclear CRTC1 and CRTC2 as well as alters regulators of their localization; 3) overexpression of CRTC1 or CRTC2 increases PGC-1α promoter activity and the response is dependent on the presence of the CRE-site; and 4) overexpression of CRTC2 increases PGC-1α mRNA and protein. In addition, our results indicate that overexpression of CRTC2 is not sufficient to prevent the Dex-induced reduction in PGC-1α or the increase in atrophy-related E3 ubiquitin ligases suggesting that Dex reduces PGC-1α by mechanisms that involve both CRTC-dependent transcription and other post-transcription process(es) that is independent of CRTCs.

CRTCs were originally identified as potent activators of CRE-dependent, CREB-mediated gene expression [20,21,30]. Increases in cAMP and calcium synergistically activate CRTCs and both signals increase PGC-1α expression [18,24,31,32]. The importance of the CRE site in the CREB response has been validated by demonstrating that induction of PGC-1α transcription by CRTCs is prevented by a dominant negative CREB protein (ACREB) that cannot bind to the CRE site [19,29,33]. Consistent with these previous studies, we found that incubating myotubes with IBMX augments PGC-1α promoter activity and deletion of the promoter CRE site reduces its activity. In addition, overexpression of either CRTC1 or CRTC2 induce promoter responses similar to those in previous reports and raises the levels of PGC-1α mRNA and protein, thus demonstrating that under non-atrophic conditions, the regulation of PGC-1α by CREB/CRTC2 is normal in our model. Following Dex treatment, we observe a number of changes consistent with our hypothesis that Dex reduces PGC-1α expression, in part, by its inhibitory action on calcineurin and the consequential effects on CRTC signaling. Dex decreases nuclear CnA as well as increases nuclear SIK. Based on earlier studies by others [23,24], these changes likely explain the reduction in nuclear localization of endogenous CRTCs and consequently contribute to the decrease in PGC-1α expression.

Curiously, overexpression of CRTC2 is not sufficient to overcome the attenuating action of Dex on PGC-1α promoter activity, mRNA and protein. As indicated in the introduction, PGC-1α is tightly regulated at the transcriptional, post-transcriptional and post-translational levels with many inputs. Our data suggest that increasing the nuclear level of CRTC2 does not overcome all of the Dex-induced responses that attenuate PGC-1α expression. For example CREB interacts with CBP and other transcriptional modulators (e.g., CRTC, p300) to form complexes that impart selectivity to CREB-mediated gene expression [21]. In addition to altering the localization of the CRTCs, Dex may change the activity of one or more other proteins in the complex that interacts with the PGC-1α promoter CRE site. Dex could also simultaneously change the activity of other Cn-regulated transcription factors that participate in PGC-1α transcription (e.g., MEF2, NFAT). Another possibility is that Dex alters the cellular level of one or more microRNAs (miRs) [34,35]. miRs are short, non-coding RNAs that inhibit the translation and/or promote the degradation of specific mRNAs [36]. Previously, our lab reported that Dex alters the levels of several miRs whose targets are involved in the atrophy process (e.g. MuRF1, Atrogin-1/MAFbx, FoxO3 [37,38]). Notably, at least one of these miRs, miR23a, is down-regulated, in part, by a mechanism involving Dex-induced suppression of Cn [38]. Although untested, Dex may increase the level of an unidentified microRNA(s) that targets PGC-1α in myotubes and skeletal muscle. Such a response could explain the greater suppression of PGC-1α mRNA and protein, relative to changes in transcription, in cells treated with Dex.

In summary, our studies indicate that Dex reduces PGC-1α expression in muscle cells by several mechanisms. Although our results do not fully elucidate all of the mechanisms that frequently lower the level of coactivator in chronic systemic diseases, our study is the first to investigate the underlying cause(s) of the response. One mechanism involves dysfunctional CREB/CRTC signaling and PGC-1α transcription. In addition, Dex appears to also induce other cell responses that impact PGC-1α mRNA and protein independent of the CRTC/CREB pathway.
